# Association between P2X7 Polymorphisms and Post-Transplant Outcomes in Allogeneic Haematopoietic Stem Cell Transplantation

**DOI:** 10.3390/ijms21113772

**Published:** 2020-05-27

**Authors:** Rachel M Koldej, Travis Perera, Jenny Collins, David S Ritchie

**Affiliations:** 1 ACRF Translational Research Laboratory, Royal Melbourne Hospital, Melbourne, VIC 3050, Australia; Travis.Perera@ccdhb.org.nz (T.P.); jenny.collins@mh.org.au (J.C.); David.Ritchie@mh.org.au (D.S.R.); 2 Faculty of Medicine, Dentistry and Health Sciences, University of Melbourne, Melbourne, VIC 3010, Australia; 3 Department of Clinical Haematology, Peter MacCallum Cancer Centre and Royal Melbourne Hospital, Melbourne, VIC 3000, Australia

**Keywords:** P2X7, polymorphism, haematopoietic stem cell transplantation

## Abstract

Allogeneic stem cell transplantation (alloSCT) is a highly effective treatment method for haematologic malignancies. However, infection of acute organ dysfunction and graft versus host disease (GVHD) impact negatively on patient outcomes. Pre-transplant conditioning regimes are associated with high levels of immunogenic cell death and the release of extracellular ATP, which binds to the P2X7 receptor. It has been proposed that signaling through the P2X7 receptor may lead to activation of downstream effectors that influence alloSCT outcome. In this study, we examined the effect of gain-of-function (GOF) or loss-of-function (LOF) P2X7 Single Nucleotide Polymorphisms (SNP) in 453 paired alloSCT donors and recipients and correlated their presence or absence to the major post-transplant outcomes of acute GVHD, relapse free survival and overall survival. The allelic frequency of P2X7 SNP in recipients and donors was not different from those SNP for which there is published population data. The LOF SNP Glu496Ala was overrepresented in recipients who did not develop severe acute GVHD and was associated with improved overall survival in rare homozygous recipients, whereas the LOF SNP Ile568Asn was more common in patients with grade 1–4 GVHD but lost statistical association in patients with grade 2–4 aGVHD, and was associated with reduced overall survival in heterozygotes due to an excess of infection-related deaths. The GOF variant haplotype (homozygous Gln460Arg-Ala348Thr) had no impact on post-alloSCT outcomes. Overall, our data indicate that allelic variations in recipients or donors occurs at the same frequency as the general population and may have a minor, but clinically nominal, impact on post-alloSCT outcomes.

## 1. Introduction

Allogeneic stem cell transplantation (alloSCT) is a curative therapy in a range of malignant and non-malignant blood conditions. The success of alloSCT is limited by the onset of opportunistic infections, development of graft versus host disease (GVHD) or conversely by limitations in the strength of the graft versus tumour (GVT) effect leading to disease progression or relapse [1]. As post-transplant outcomes are largely immunologically mediated, extensive exploration has been undertaken to identify determinants of donor immune activation and/or reconstitution post-transplant [2,3,4,5]. The influence of recipient immunological function on subsequent donor immune activation has been less studied. Similarly, while post-alloSCT biomarkers for GVHD outcome [6,7,8] and clinical risk [9] have been identified in large cohorts, there are no clinically utilised recipient pre-transplant biomarkers for post-transplant outcomes. Currently, the selection of patients suitable for alloSCT is largely dependent on clinical assessment and quantification of comorbidity indices for estimates of transplant-related mortality (TRM) and disease risk indices (DRI) for risk of relapse [10].

In the process of allogeneic transplantation, conditioning-regimen induced tissue inflammation, cellular apoptosis and presentation of recipient minor histocompatibility antigens prime donor T cells for future onset of GVHD [11]. Similarly, donor T cell priming against tumour antigens in the immediate post-transplant period may promote the formation of GVT immunity.

Donor T cell activation, and therefore the priming of either GVHD or GVT effects is dependent on the presence of a pro-inflammatory microenvironment and presentation of minor histocompatibility tissue antigens [12]. Molecules responsible for sensing tissue damage, modifying inflammatory responses and removing or promoting the presentation of tissue antigens by antigen-presenting cells are likely to, therefore, modify subsequent donor T cell responses. Murine models of acute GVHD (aGVHD) have helped identify the inflammatory pathways involved in the intestinal tract and skin and shown a major role for the P2X7 receptor in immune and haemopoietic cells of the graft recipient [13].

The purinergic receptor P2X7 is ubiquitously expressed in tissues and organs of the body, including immune and haemopoietic cells, with particularly high expression found on cells of the monocyte and macrophage lineage [14]. Activation of P2X7 by its physiological agonist, ATP, opens a cation-selective maxi-channel, which allows a large K+ efflux leading to the assembly of the NLRP3 inflammasome in monocyte and macrophages [15,16]. The channel is also permeable to fluorescent cation dyes, and dye uptake studies have shown that the P2X7 receptor function varies widely between individuals in the population [17]. A large part of this variation results from functional polymorphisms in P2X7 which can either lead to loss of function (e.g., Arg307Gln [18], Glu496Ala [19]) or gain of function (e.g., Ala348Thr [20]) and this functional variation directly affects downstream signaling from the receptor to the NLRP3 inflammasome which in turn affects the processing and release of pro-inflammatory cytokines, notably IL-1β and IL-18 [20,21,22]. Thus, activation of P2X7 on immune cells infiltrating the intestinal tract and skin provide a direct stimulus for assembly of the NLRP3 inflammasome, the activity of which may regulate aGVHD [23]. Genetic knock out of P2X7 has been shown to reduce the severity of aGVHD in mouse models of alloSCT and by the use of chimeric mice, it was shown that this protective effect was only present when P2X7 was ablated from the recipient’s hemopoietic system [13]. The human P2X7 receptor is notable for the dozen or more polymorphic variants that alter receptor function in the Caucasian population (reviewed by [14]). Loss of function polymorphisms present in around one quarter of the population can be present on one or both alleles of the P2X7 gene, and homozygosity produces severe hypofunction of this receptor with a predisposition to infection with obligate intracellular pathogens [24,25]. The main gain of function variant, Ala348Thr, has been shown to lie in a haplotype block spanning exons 11 to 13 of the P2X7 gene [24]. Five functional variants lie within the block, three of which decrease function and one, Glu460Arg with only small effects. However, the haplotype 348Thr-460Arg (haplotype 4) exerts the strongest effect with a five-fold increase in function, whereas 348Thr-460Gln (haplotype 2) increases function only two-fold [24].

In light of the potential for P2X7 to play a central role in modifying immune responses post alloSCT we investigated if P2X7 SNP in allogeneic transplant recipients and their paired donors influence post alloSCT outcomes in a large cohort of patients transplanted at our centre.

## 2. Results

### 2.1. Allelic Frequency

In total, we analysed 522 donor and 644 recipient samples from patients that underwent alloSCT at our centre over a 16-year period. The alleleic frequency was compared between recipients and donors and with previously published cohorts (Table 1). We identified no difference in SNP minor allele frequency (MAF) between donor or recipients, and the MAF was comparable to previously published frequencies in the general population.

### 2.2. Paired Cohort Patients

A subgroup of 453 paired donor and recipient samples (906 samples in total) were identified from our main cohort and analysed to explore the impact of either recipient or donor P2X7 SNP on clinical outcomes of allogeneic transplantation. Details of patient and transplant characteristics are shown in Table 2.

Clinical outcome data for correlation with SNP findings were obtained from the Bone Marrow Transplant database of the Royal Melbourne Hospital. Acute GVHD was defined within this database by the onset of typical clinical symptoms and signs and confirmed where clinically applicable by tissue biopsy of affected areas. Grading of GVHD was undertaken using NIH consensus guidelines [26]. Patients relapsing prior to the onset of aGVHD were excluded from the aGVHD analysis but remained assessable for Relapse Free Survival (RFS) and Overall Survival (OS) analyses. RFS and OS were calculated from day zero of the transplant, and the time of documented relapse, date of last follow up or date of death.

### 2.3. Single SNP Associations with Transplant Outcome

Pre-transplant DNA samples from donors and recipients were analysed by SNP array for the presence of 16 previously published SNP in P2X7 [17,18,19,27,28]. Five SNP (Arg117Trp, Glu186Lys, Leu191Pro, His521Gln and null allele rs35933842) occurred at very low frequencies and were excluded from further analysis. All other SNP were analysed for their effect on aGVHD, RFS and OS. Details of all analyses outcomes can be found in Supplementary Materials Tables S2–S4.

**Figure 1:**
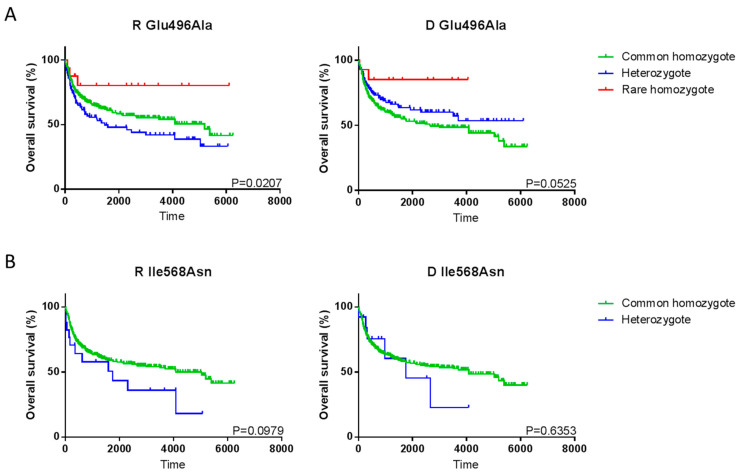
Association between Recipient (R) or Donor (D) Glu496Ala and Ile568Asn and overall survival using the Kaplan–Meier method. (**A**) Glu496Ala recipients (left, common homozygote n = 307, heterozygote = 129, rare homozygote = 16. One recipient SNP analysis failed and was excluded from analysis) and donors (right, common homozygote n = 302, heterozygote = 137, rare homozygote = 14). (**B**) Ile568Asn recipients (left, common homozygote n = 435, heterozygote = 17. 1 recipient SNP analysis failed and was excluded from analysis) and donors (right, common homozygote n = 437, heterozygote = 13. One donor was rare heterozygote and was excluded from analysis. Two donors SNP analysis failed and were excluded from analysis).

Only two SNP could be associated with any of the post-transplant clinical outcomes. The SNP Ile568Asn and Glu496Ala were associated with an increased or decreased occurrence of aGVHD, respectively (Table 3). Recipient Glu496Ala was associated with significantly reduced aGVHD only when categorizing patients into grade 0–1 vs. grade 2–4 aGVHD. Glu496Ala was also associated with significantly improved OS in rare homozygous recipients compared to either common homozygotes or heterozygotes (*P* = 0.0207) and demonstrated the same trend when the SNP was present in donors, though statistical significance was not reached (*P* = 0.0525) (Figure 1A). Recipient Ile568Asn was associated with increased rates of aGVHD (grade 0 vs. grade 1–4 only) and while there was a trend to decreased survival in recipient Ile568Asn heterozygotes, this was not statistically significant (*P* = 0.0979, HR = 2.331, 95%CI 1.267–4.289) and no such trend was seen in donors (Figure 1B).

### 2.4. P2X7 Haplotype Associations with Transplant Outcome

The impact of P2X7 has been previously suggested to occur when present in combination as a haplotype. Fourteen recipients and seven donors were found to be homozygous for both Gln460Arg and Ala348Thr SNP and were, therefore, were classified as homozygous P2X7-4 haplotype. There were an additional 60 recipients and 45 donors who were homozygous Ala348Thr but not Gln460Arg, who were classified as homozygous P2X7-2 haplotype. In the analyses of these haplotypes against the post-transplant outcomes of aGVHD, RFS and OS, we were unable to demonstrate any significant associations (Supplementary Materials Figure S1 and Table S5).

## 3. Discussion

Studies to identify markers of patient outcomes in alloSCT have often focused on the biology of donor cells. Conversely, recipient immunology has been infrequently studied as a determinant of transplant outcome, although more recently, circulating biomarkers linked to recipient tissue damage have been used to prognostically determine survival in aGVHD [29]. Factors such as P2X7 activation might alter the clearance of tissue antigens and limit the speed and activation status of donor T cells. In this analysis, we have, therefore, specifically examined the role of multiple P2X7 SNP in alloSCT recipients and correlated to post-transplant outcomes.

Of the 16 SNP analysed from over 450 recipient and donor pairs, we found that only Ile568Asn and Glu496Ala were associated with changes in aGVHD risk and only when comparing absent or grade 1 GVHD with higher grades. These 2 LOF P2X7 SNP occur at a lower frequency than GOF SNP, suggesting that reduced function of P2X7 may be of a greater immunological consequence than increased function in the context of alloSCT. Most interestingly, the presence of these two LOF SNP in alloSCT recipients had opposite impacts on alloSCT outcomes, which may be related to their underlying biology.

P2X7 loss of function is known to influence aGVHD biology in mouse models. The P2X7 receptor antagonist Brilliant Blue G has been shown to reduce GVHD onset, improve liver function [30] and reduce interferon-γ in mouse models of GVHD [31]. In P2X7^-/-^ recipient mice transplanted with P2X7^+/+^ bone marrow aGVHD developed at a reduced rate compared to P2X7^+/+^ recipient mice transplanted with P2X7^+/+^ bone marrow [13]. Furthermore, donor P2X7 activity in leukocytes does not affect the development of aGVHD in a humanized NSG model [32]. It has been proposed that ATP release from injured tissue engages with recipient APCs leading to increased CD80/86 expression, co-stimulation of donor CD4^+^ cells and increased interferon-γ expression promoting aGVHD onset [13]. While a previous small analysis of P2X7 SNP Glu496Ala homozygosity on transplant outcome (n = 125) showed an overall poorer OS post alloSCT, no association with aGVHD was found [33] and this association with OS was unable to be replicated in a larger study of 2888 recipients [34]. We also could not replicate this finding in our study, and in fact, identified an opposite association, which may reflect differences in sample size, patient ethnicity and/or treatment regimes. In agreement with the P2X7^−/−^ mouse model data, our study demonstrated that LOF Glu496Ala on recipient haematopoietic cells is associated with reduced rates of aGVHD and increased OS.

The Ile568Asn SNP is related to loss of phagocytic-function and in this study resulted in an increased incidence and rate of aGVHD [27]. The Ile568Asn SNP is known to prevent the trafficking of P2X7 to the surface membrane, and heterozygosity for this allele in mononuclear cells confers reduced P2X7 surface expression and a corresponding reduction in phagocytosis of apoptotic debris [27]. A possible consequence of this SNP may be an increased amount of free recipient antigen available for presentation to donor T cells, thus resulting in priming of allo-aggressive T cell clones and increased incidence of aGVHD. In this cohort of patients, those who developed aGVHD either died from organ dysfunction related to the aGVHD or subsequently developed cGVHD and died from fatal infection complicating its therapy. This suggests that not only are recipients with Ile568Asn more likely to develop aGVHD, but they are also more likely to develop fatal infections possibly due to immunosuppression-associated worsening of the already impaired phagocytic function that is associated with Ile568Asn.

The association between P2X7 SNP inheritance, haematologic malignancies and patient outcome has been previously studied to a limited extent in small cohorts. In Multiple Myeloma [35], loss of function P2X7 SNP has been associated with disease risk. Conversely, higher P2X7 expression has been shown in patients with relapsed CLL [36] and in paediatric Acute Myeloid Leukaemia (AML) [37]. Increased P2X7 mRNA expression in leukaemic blasts has been associated with reduced remission rates following induction chemotherapy for AML [38]. We did not find an overrepresentation of any P2X7 SNP with any diagnostic group, relapse risk or disproportionate distribution in the 453 patients with haematological malignancy that we examined. Indeed, MAF in both patients and their donors were comparable to published allele rates in the general population, suggesting that clinical application of P2X7 SNP genotyping in alloSCT patients is not warranted.

This study is potentially limited by the low allelic frequency of most P2X7 polymorphisms in the general population, which did not change in alloSCT recipients, and therefore analysis in a larger cohort could be warranted.

Our findings contribute to the understanding of P2X7 biology and how that relates to the complex patient outcomes post-alloSCT. Despite prior recognition of the P2X7 pathway in immunological and phagocytic functions and our own identification of differences in outcome in patients with two LOF SNP, we could not demonstrate an effect of a single SNP or haplotype in donors or recipients that could be meaningfully applied to pre- or post-transplant decision making regarding the application of alloSCT.

## 4. Materials and Methods

### 4.1. Patient Samples

This study was conducted in accordance with the Declaration of Helsinki, and the protocol was approved by the Melbourne Health Human Research Ethics Committee (HREC/14/MH/373). Patients who had undergone allogeneic bone marrow transplantation with either peripheral blood or bone marrow from related or unrelated adult donors between 2002 and 2018 at Royal Melbourne Hospital and had pre-transplant peripheral blood DNA samples available were eligible for this study.

### 4.2. P2X7 Polymorphism Analysis

All samples were analysed using Agena Bioscience MassARRAY^®^ and iPLEX GOLD chemistry for the presence of 16 SNP by the Australian Genomics Research Facility (St Lucia, QLD, Australia). The list of primer sequences used is shown in Supplementary Materials Table S1.

### 4.3. Statistical Analysis

Analysis of correlation of clinical outcome and individual SNP was performed using Fisher’s exact test in GraphPad Prism 6. The probabilities of OS and RFS were calculated and plotted using the Kaplan-Meier method in GraphPad Prism 6. OS and RFS P values were determined using the Log-rank (Mantel-Cox) test, and the median survival hazard ratio and 95% confidence interval are reported and were able to be calculated.

## Figures and Tables

**Table 1:** P2X7 Single Nucleotide Polymorphism (SNP) Minor Allele Frequency (MAF).

dbSNP ID	Amino Acid Change	Effect on Function	Published MAF ^1^	Recipient MAF	Donor MAF
rs28360445	Arg117Trp	loss	nd	0.003	0.002
rs28360447	Gly150Arg	loss	0.018	0.016	0.017
rs28360451	Glu186Lys	loss	nd	0.000	0.000
rs28360452	Leu191Pro	loss	nd	0.000	0.000
rs7958311	Arg270His	loss	0.255	0.255	0.291
rs7958316	Arg276His	loss	0.02	0.023	0.031
rs28360457	Arg307Gln	loss	0.013	0.012	0.018
rs3751143	Glu496Ala	loss	0.175	0.185	0.180
rs1653624	Ile568Asn	loss	0.029	0.019	0.015
rs35933842	-	loss	0.008	0.008	0.007
rs17525809	Val76Ala	partial loss	0.062	0.069	0.089
rs2230911	Thr357Ser	partial loss	0.083	0.099	0.084
rs2230912	Gln460Arg	partial loss	0.170	0.165	0.144
rs2230913	His521Gln	neutral	0.02	0.001	0.001
rs208294	His155Tyr	gain	0.439	0.470	0.447
rs1718119	Ala348Thr	gain	0.4	0.385	0.367

^1^ From [17]. nd = not determined.

**Table 2:** Characteristics of the patients with paired samples included in this study.

Characteristics (Total n = 453)	n	%
Recipient Age, median (range), years	47	(16–73)
Gender		
F	190	(41.9%)
M	263	(58.1%)
Stem cell source		
BM	49	(10.8%)
PB	404	(89.2%)
Donor		
RD	247	(54.5%)
MUD	206	(45.5%)
Conditioning		
MAC	357	(78.8%)
RIC	96	(21.2%)
Disease risk index		
Low	77	(16.1%)
Intermediate	257	(56.7%)
High	73	(16.1%)
Very high	12	(2.6%)
Not determined	34	(7.5%)
Diagnosis		
AML	159	(35.1%)
ALL	63	(13.9%)
MDS	44	(9.7%)
MM	28	(6.2%)
FL	27	(6.0%)
MF	22	(4.9%)
CLL	20	(4.4%)
CML	17	(3.8%)
HL	15	(3.3%)
Other	58	(12.8%)

Female (F), Male (M), Bone Marrow (BM), Peripheral Blood (PB), Related Donor (RD), Matched Unrelated Donor (MUD), Myeloablative Conditioning (MAC), Reduced Intensity Conditioning (RIC), Acute Myeloid Leukemia (AML), Acute Lymphoblastic Leukaemia (ALL), Myelodysplastic Syndrome (MDS), Multiple Myeloma (MM), Follicular Lymphoma (FL), Chronic Lymphocytic Leukaemia (CLL), Chronic Myeloid Leukaemia (CML), Hodgkin Lymphoma (HL).

**Table 3:** Summary of significant SNP identified.

Outcome	Donor or Recipient	SNP	MAF		*p* Value	RR	95% CI
			Unaffected	Affected			
aGVHD (g0 vs. g1–4)	Recipient	Ile568Asn	0.01	0.04	0.0173	1.027	1.003–1.051
aGVHD (g0–1 vs. g2–4)	Recipient	Glu496Ala	0.19	0.13	0.0457	0.9267	0.8683–0.989

## Supplementary Materials

Supplementary Materials can be found at https://www.mdpi.com/1422-0067/21/11/3772/s1.
