# Supplementary material for: Association between P2X7 Polymorphisms and Post-Transplant Outcomes in Allogeneic Haematopoietic Stem Cell Transplantation
Source: Int J Mol Sci. 2020 May 27;21(11):3772. doi: 10.3390/ijms21113772 (PMC7312926; doi:10.3390/ijms21113772)
Supplement: Supplementary file 1 [file ijms-21-03772-s001.pdf]

**Supplementary Materials**

**Supplementary Table S1**: P2X7 primer sequences

| SNP_ID | PCR Primer 1 | PCR Primer 2 | Extension Primer |
|---|---|---|---|
| rs28360445 | ACGTTGGATGACTCCAGGTCCCCTCCTTAC | ACGTTGGATGGGAACTCTTTCTTCGTGATG | CCTCGGGACACAACC |
| rs28360447 | ACGTTGGATGTTCTACGATGCTTTGACCCC | ACGTTGGATGCAGAGACTTCACAGGTCTTC | CCCTATAGGAATTCAGACC |
| rs28360451 | ACGTTGGATGCAGGCCTGCTCTCTTGAAC | ACGTTGGATGCTTACGTGGTGTAGTTGTGG | CCTGCTCTCTTGAACAGTGCC |
| rs28360452 | ACGTTGGATGGCACTTACGTGGTGTAGTTG | ACGTTGGATGTGAACAGTGCCGAAAACTTC | AGTCGATATTGTTCTTGATG |
| rs7958311 | ACGTTGGATGGTCGTCAAGGCGAAGGAAAC | ACGTTGGATGTTGAGATCTACTGGGACTGC | IGGAAACTGTATTTGGGA |
| rs7958316 | ACGTTGGATGAGGGTACAAGGACACGTTGG | ACGTTGGATGTAGACCGTTGGTTCCATCAC | TCTTGTCGTCAAGGCGA |
| rs28360457 | ACGTTGGATGCTTCCTACAGATACGCCAAG | ACGTTGGATGTCAAAACGGATCCCGAAGAC | ACGGAAAACAATGTTGAGAAAC |
| rs3751143 | ACGTTGGATGAACAGCTCTGAGGTGGTGAT | ACGTTGGATGATCTCAACTCCCTGAGAGCC | TTCCGGCAGCACAGC |
| rs1653624 | ACGTTGGATGACTGTGCCTACAGGTGCTAC | ACGTTGGATGTCTTTCCGGATCCTCCAGC | TGGCTGACTTTGCCA |
| rs35933842 | ACGTTGGATGTTTCTGTGCTGTCGGCCACT | ACGTTGGATGGGTTCTTCCACGTGATCATC | CCCCAGATCCCACTTA |
| rs17525809 | ACGTTGGATGTCATCAGTTCTGTGCACACC | ACGTTGGATGCACTGTGCACCAACTTCTTC | AGGTGAAAGAGGAGATCG |
| rs2230911 | ACGTTGGATGCTGTGTTCATCGACTTCCTC | ACGTTGGATGGGGATAAATATGGGAGCGAC | CGACTTCCTCATCGACA |
| rs2230912 | ACGTTGGATGTATCCCTGGATCTAGGAGTC | ACGTTGGATGTCCATGACACACCCCCGATT | CCTCCTTTCTAAGCAGC |
| rs2230913 | ACGTTGGATGTTGGTGGAATCCACATCCAG | ACGTTGGATGAGCTGTTCAGGAAGCTGGTC | GAGGAACTGCAGGAC |
| rs208294 | ACGTTGGATGCAGAGACTTCACAGGTCTTC | ACGTTGGATGTTCTACGATGCTTTGACCCC | CTTCTGGTTCCCTTCAT |
| rs1718119 | ACGTTGGATGTGTCGATGAGGAAGTCGATG | ACGTTGGATGGAGAAGGAAGTGACTAACGC | GAGGAAGTCGATGAACACAG |

**Supplementary Table S2**: MAF correlation to aGVHD grade

Recipient

| Polymorphism | MAF | | | | | MAF | | | | |
|---|---|---|---|---|---|---|---|---|---|---|
| | grade 0 | grade 1-4 | P value | RR | 95% CI | grade 0-1 | grade 2-4 | P value | RR | 95% CI |
| Gly150Arg | 0.01 | 0.02 | 0.388 | 1.008 | 0.9894-1.027 | 0.02 | 0.01 | 0.7432 | 0.9937 | 0.9769-1.011 |
| Arg270His | 0.24 | 0.29 | 0.1415 | 1.066 | 0.9790-1.161 | 0.25 | 0.29 | 0.2661 | 1.058 | 0.9591-1.168 |
| Arg276His | 0.02 | 0.02 | 0.799 | 0.9952 | 0.9766-1.014 | 0.02 | 0.00 | 0.1416 | 0.981 | 0.6991-0.9961 |
| Arg307Gln | 0.02 | 0.01 | 1 | 0.9974 | 0.9810-1.014 | 0.02 | 0.01 | 0.7431 | 0.9935 | 0.9769-1.01 |
| **Glu496Ala** | 0.18 | 0.17 | 0.5796 | 0.981 | 0.9201-1.046 | 0.19 | 0.13 | **0.0457** | **0.9267** | **0.8683-0.989** |
| **Ile568Asn** | 0.01 | 0.04 | **0.0173** | **1.027** | **1.003-1.051** | 0.02 | 0.03 | 0.2454 | 1.014 | 0.9878-1.041 |
| Val76Ala | 0.07 | 0.09 | 0.2763 | 1.023 | 0.9811-1.066 | 0.07 | 0.10 | 0.1634 | 1.033 | 0.9827-1.086 |
| Thr357Ser | 0.10 | 0.09 | 0.7177 | 0.9885 | 0.9456-1.033 | 0.09 | 0.10 | 0.6826 | 1.012 | 0.9599-1.067 |
| Gln460Arg | 0.17 | 0.14 | 0.2449 | 0.9622 | 0.9077-1.02 | 0.17 | 0.12 | 0.1256 | 0.9471 | 0.8901-1.008 |
| His155Tyr | 0.45 | 0.46 | 0.7746 | 1.022 | 0.8980-1.162 | 0.47 | 0.43 | 0.2582 | 0.916 | 0.7971-1.053 |
| Ala348Thr | 0.40 | 0.38 | 0.4658 | 0.9621 | 0.8727-1.061 | 0.39 | 0.40 | 0.804 | 1.021 | 0.8970-1.163 |

Donor

| Polymorphism | MAF | | | | | MAF | | | | |
|---|---|---|---|---|---|---|---|---|---|---|
| | grade 0 | grade 1-4 | P value | RR | 95% CI | grade 0-1 | grade 2-4 | P value | RR | 95% CI |
| Gly150Arg | 0.02 | 0.02 | 1 | 0.9973 | 0.9790-1.016 | 0.02 | 0.02 | 0.7691 | 1.002 | 0.9802-1.025 |
| Arg270His | 0.28 | 0.29 | 0.7534 | 1.016 | 0.9297-1.11 | 0.28 | 0.29 | 1 | 1.003 | 0.9080-1.108 |
| Arg276His | 0.03 | 0.03 | 1 | 0.9982 | 0.9740-1.023 | 0.03 | 0.02 | 0.8161 | 0.992 | 0.9666-1.018 |
| Arg307Gln | 0.02 | 0.02 | 1 | 1.001 | 0.9811-1.02 | 0.02 | 0.02 | 1 | 1.001 | 0.9785-1.023 |
| Glu496Ala | 0.18 | 0.18 | 0.8543 | 0.9914 | 0.9289-1.058 | 0.19 | 0.16 | 0.4682 | 0.9707 | 0.9042-1.042 |
| Ile568Asn | 0.02 | 0.02 | 0.785 | 1.004 | 0.9852-1.024 | 0.01 | 0.03 | 0.1235 | 1.017 | 0.9911-1.044 |
| Val76Ala | 0.08 | 0.10 | 0.2064 | 1.028 | 0.9833-1.075 | 0.08 | 0.11 | 0.114 | 1.042 | 0.9869-1.1 |
| Thr357Ser | 0.08 | 0.08 | 1 | 1.001 | 0.9600-1.044 | 0.08 | 0.10 | 0.4645 | 1.018 | 0.9687-1.071 |
| Gln460Arg | 0.15 | 0.13 | 0.3645 | 0.9733 | 0.9209-1.029 | 0.15 | 0.13 | 0.5677 | 0.9779 | 0.9195-1.040 |
| His155Tyr | 0.47 | 0.40 | 0.0635 | 0.8875 | 0.7872-1.001 | 0.46 | 0.40 | 0.1702 | 0.9039 | 0.7917-1.032 |
| Ala348Thr | 0.36 | 0.37 | 0.8238 | 1.015 | 0.9120-1.129 | 0.36 | 0.37 | 0.8673 | 1.013 | 0.8982-1.144 |

RR = Relative Risk, CI = Confidence Interval

**Supplementary Table S3**: P2X7 SNP correlation to RFS

Recipient

| Polymorphism | P value | Hazard Ratio | 95% CI |
|---|---|---|---|
| Gly150Arg | 0.0958 | 2.236 | 0.8673-5.764 |
| Arg270His* | 0.7383 | | |
| Arg276His | 0.6932 | 0.8369 | 0.3455-2.057 |
| Arg307Gln | 0.4300 | 0.6531 | 0.2267-1.881 |
| Glu496Ala* | 0.5178 | | |
| Ile568Asn | 0.8231 | 1.114 | 0.4329-2.866 |
| Val76Ala | 0.4364 | 0.8232 | 0.5044-1.344 |
| Thr357Ser* | 0.4972 | | |
| Gln460Arg* | 0.1942 | | |
| His155Tyr* | 0.2926 | | |
| Ala348Thr* | 0.4755 | | |

*Analysis of 3 groups (common homozygote, heterozygote and rare homozygote). Hazard ratio and 95% CI unable to be calculated.

Donor

| Polymorphism | P value | Hazard Ratio | 95% CI |
|---|---|---|---|
| Gly150Arg | 0.8160 | 0.8944 | 0.3494-2.290 |
| Arg270His* | 0.7584 | | |
| Arg276His | 0.7054 | 0.8634 | 0.4032-1.849 |
| Arg307Gln | 0.6313 | 0.8024 | 0.3266-1.972 |
| Glu496Ala* | 0.4417 | | |
| Ile568Asn | 0.8172 | 1.135 | 0.3874-3.326 |
| Val76Ala | 0.6779 | 1.099 | 0.7041-1.715 |
| Thr357Ser* | 0.6009 | | |
| Gln460Arg* | 0.1961 | | |
| His155Tyr* | 0.6454 | | |
| Ala348Thr* | 0.2194 | | |

*Analysis of 3 groups (common homozygote, heterozygote and rare homozygote). Hazard ratio and 95% CI unable to be calculated.

**Supplementary Table S4**: P2X7 SNP correlation to OS

Recipient

| Polymorphism | P value | Hazard Ratio | 95% CI |
|---|---|---|---|
| Gly150Arg | 0.4569 | 1.371 | 0.5971-3.147 |
| Arg270His* | 0.7105 | | |
| Arg276His | 0.3761 | 3.039 | 1.606-5.751 |
| Arg307Gln | 0.1206 | 0.9980 | 0.4103-2.428 |
| **Glu496Ala*** | **0.0207** | | |
| Ile568Asn | 0.0979 | 2.331 | 1.267-4.289 |
| Val76Ala | 0.9610 | 0.9993 | 0.6633-1.505 |
| Thr357Ser* | 0.8168 | | |
| Gln460Arg* | 0.7960 | | |
| His155Tyr* | 0.7497 | | |
| Ala348Thr* | 0.5088 | | |

*Analysis of 3 groups (common homozygote, heterozygote and rare homozygote). Hazard ratio and 95% CI unable to be calculated.

Donor

| Polymorphism | P value | Hazard Ratio | 95% CI |
|---|---|---|---|
| Gly150Arg | 0.3724 | 4.919 | 2.422-9.993 |
| Arg270His* | 0.1517 | | |
| Arg276His | 0.8939 | 1.046 | 0.5419-2.018 |
| Arg307Gln | 0.4111 | 0.9993 | 0.5113-1.953 |
| Glu496Ala* | 0.0525 | | |
| Ile568Asn | 0.6353 | 2.329 | 1.032-5.256 |
| Val76Ala | 0.8184 | 0.7871 | 0.5398-1.148 |
| Thr357Ser* | 0.4657 | | |
| Gln460Arg* | 0.3545 | | |
| His155Tyr* | 0.6456 | | |
| Ala348Thr* | 0.7037 | | |

*Analysis of 3 groups (common homozygote, heterozygote and rare homozygote). Hazard ratio and 95% CI unable to be calculated.

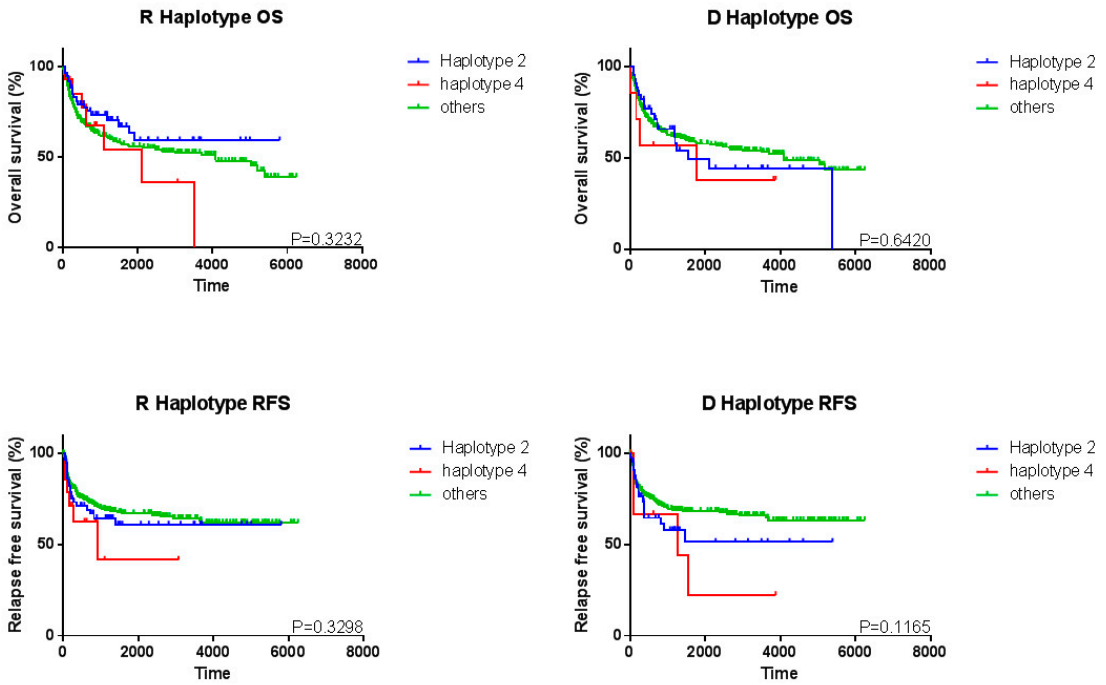


**Supplementary Figure S1: Association between recipient (R) and donor (D) haplotype and overall survival (OS) and relapse free survival (RFS).** Patients were classified as haplotype 2 (homozygous Ala348Thr), haplotype 4 (homozygous Gln460Arg and Ala348Thr) or other. Proportions of OS and RFS were calculated using Kaplan-Meier method.

**Supplementary Table S5**: Correlation of aGVHD grade to haplotype

| | Haplotype 2 | | Haplotype 4 | | Others | | P value* |
|---|---|---|---|---|---|---|---|
| | Grade 0 | Grade 1-4 | Grade 0 | Grade 1-4 | Grade 0 | Grade 1-4 | |
| Recipient | 41 | 18 | 10 | 3 | 234 | 129 | 0.5118 |
| Donor | 27 | 17 | 6 | 1 | 245 | 133 | 0.4561 |

| | Haplotype 2 | | Haplotype 4 | | Others | | P value* |
|---|---|---|---|---|---|---|---|
| | Grade 0-1 | Grade 2-4 | Grade 0-1 | Grade 2-4 | Grade 0-1 | Grade 2-4 | |
| Recipient | 45 | 14 | 11 | 2 | 280 | 83 | 0.8041 |
| Donor | 31 | 13 | 6 | 1 | 291 | 87 | 0.5293 |

*analysed using Chi square test. Any patients who relapsed prior to aGVHD or had an undetermined aGVHD grade were excluded from analysis.
